# Psychopathological dimensions in familial amyloid polyneuropathy patients

**DOI:** 10.1186/1750-1172-10-S1-O27

**Published:** 2015-11-02

**Authors:** Alice Lopes, Alexandra Sousa, Isabel Fonseca, Margarida Branco, Carla Rodrigues, Paula Freitas, Teresa Coelho

**Affiliations:** 1 Centro Hospitalar do Porto, Corino de Andrade Unit, 4099-001, Porto, Portugal; 2 Universidade do Porto, Instituto de Ciências Biomédicas Abel Salazar, 4050-313, Porto, Portugal

## Introduction

There are very few studies about psychopathology in familial amyloid polyneuropathy patients or in asymptomatic carriers. In our clinical experience in a psychiatric and psychological consultation, we mostly see patients suffering from depression and anxiety symptoms and emotional distress related to some specific, emotionally charged moments caused by the disease.

We wanted to evaluate psychopathological dimensions in the population that attends external consultation at Corino de Andrade Unit.

## Methods

The sample was composed by 211 subjects (in 110, the disease had already begun, 82 were asymptomatic carriers, and 19 had no established diagnosis); 84 were men and 127 were female. Mean ages: carriers 33.9±9.8yr, patients 37.8±8.1yr, and for subjects that had no established diagnosis, 40.9±14.0 yr. Most subjects were married or lived with a partner (67.1%) and most of them were still working; 33% were retired from work or on a sick leave.

A sociodemographic questionnaire and The Brief Symptom Inventory – BSI(Derogatis,1982, Canavarro,1999, 2007) were applied to these patients.

BSI is an inventory that evaluates 9 psychopathological dimensions (somatization, obsession-compulsion, interpersonal sensitivity, depression, anxiety, hostility, phobic anxiety, paranoid ideation, psychoticism) and includes a global symptom index (GSI).

Statistical analysis was applied (descriptive analysis, Mann-Whitney and Wilcoxon, Spearman tests).

## Results

Considering Global Symptom Index (GSI), 32.7 % of total subjects were above media for general population. When sub groups were evaluated, 25.6% of symptomatic carriers,26.3 % of subjects without established diagnosis and 39.1% of patients were above media.

All dimensions of BSI were significantly higher in the group of patients when compared with that of the carriers, with the exception of obsession-compulsion, phobic anxiety and interpersonal sensitivity. The global symptom index was significantly higher in patients (p=0.001).

When we considered differences between gender, women who were asymptomatic carriers had statistically significant more phobic anxiety (p=0.01) and almost significant interpersonal sensitivity, anxiety and depression.

In the group of patients, almost all dimensions scored significantly higher for women, with the exception of somatization (p=0.065).

In the group of patients, all dimensions and GSI (rho=0.33, p=0.002) had positive correlations with years of disease, except for interpersonal sensitivity.

In patients, only the hostility dimension had positive correlation (rho=-0.234, p=0.031).

## Conclusions

A high number of FAP patients have psychopathological symptoms and also asymptomatic carriers, have scores above those for general population, in a significant number.

The group of patients are at higher risk for most of the psychopathological dimensions.

Sick women are more vulnerable to psychological distress, and as time goes by, patients may have more psychic problems.

